# Robust emotion recognition for complex environments: ChildEmoNet model based on DETR-ResNet50 cascaded architecture

**DOI:** 10.1371/journal.pone.0332130

**Published:** 2025-09-18

**Authors:** Zhang Shanshan, Sha Yanlin, Loy Chee Luen

**Affiliations:** 1 Department of Early Childhood Education, Faculty of National Child Development Research Centre, Universiti Pendidikan Sultan Idris, Perak, Malaysia; 2 Music Department, Faculty of Creative Arts, Universiti Malaya, Kuala Lumpur, Malaysia; 3 Department of Early Childhood Education, Faculty of Human Development, Universiti Pendidikan Sultan Idris, Perak, Malaysia; Universiti Tunku Abdul Rahman, MALAYSIA

## Abstract

Emotion recognition faces significant challenges in complex real-world environments, particularly under facial occlusion conditions that severely impact traditional deep learning approaches. This research proposes ChildEmoNet, a novel cascaded emotion recognition framework that strategically integrates Detection Transformer (DETR) for robust multi-person detection with ResNet50 for discriminative feature extraction. The primary contributions include the development of a cascaded DETR-ResNet50 architecture that addresses both detection and classification challenges simultaneously, enhanced robustness mechanisms specifically designed for facial occlusion scenarios, and comprehensive evaluation across both categorical and dimensional emotion recognition tasks. Extensive experiments on the OMG Emotion Dataset demonstrate the effectiveness of this integration: the proposed model achieves an AUC of 0.93 in standard emotion classification tasks, maintains 79% recognition accuracy under 30% facial occlusion conditions, and attains concordance correlation coefficients (CCC) of 0.52 and 0.46 for valence and arousal prediction, respectively. The experimental validation confirms the crucial role of the DETR module in processing multi-person scenarios and the effectiveness of ResNet50 in feature extraction, demonstrating superior performance across complex environmental conditions including varying lighting, face orientations, and partial occlusions. Compared with traditional methods, this cascaded architecture shows remarkable robustness under challenging real-world conditions. This research advances emotion computing technology by providing a robust solution for emotion recognition applications in complex environments where conventional approaches exhibit significant performance degradation.

## 1 Introduction

### 1.1 Background

In early childhood education, music serves as a unique teaching medium with important roles in promoting cognitive development, emotional expression, and social abilities. Children’s perception and understanding of music are primarily manifested through emotional responses, which are often more direct and authentic than verbal expressions [1,2]. However, for a long time, early childhood music education has mainly relied on teachers’ experience and fixed teaching plans, lacking scientific analysis and responses to children’s immediate emotional feedback [3,4]. With the development of computer vision and emotion computing technologies, it has become possible to introduce advanced emotion recognition algorithms into early childhood music education [5,6], which provides new ideas for addressing the lack of personalization and real-time interaction in traditional music education.

Traditional early childhood music teaching models have many limitations. Teachers find it difficult to simultaneously and accurately capture and analyze the emotional changes of each child in the classroom; preset teaching content cannot be dynamically adjusted according to children’s real-time emotional states; existing teaching systems lack scientific emotional data collection and analysis mechanisms, resulting in highly subjective teaching effect evaluations [7,8]. These problems affect children’s participation and learning effectiveness in musical activities, especially for children with weaker emotional expression abilities or introverted personalities [9,10]. Therefore, researching and developing an algorithm that accurately recognizes children’s emotional responses is of great significance for enhancing teachers’ ability to perceive children’s emotions and improving targeted music teaching.

Multimodal emotion computing technology provides innovative auxiliary tools for early childhood music education. Computer vision technology can identify and analyze children’s facial expressions in musical activities in real-time, providing objective emotional data support to teachers, assisting them in more accurately understanding children’s emotional responses and needs. This research aims to explore the application value of facial expression recognition algorithms in early childhood music education and propose an emotion recognition method suitable for children’s facial features to help teachers better grasp children’s emotional changes during musical activities, thereby optimizing teaching strategies and content selection.

### 1.2 Related work

Recent advances in artificial intelligence have driven significant progress in emotion recognition research. Deep learning algorithms demonstrate strong performance in facial expression recognition by extracting and analyzing facial features to achieve high-precision emotion classification [11]. However, most existing approaches are evaluated under controlled laboratory conditions and lack the robustness required for complex real-world applications.

Current emotion recognition technologies span multiple modalities but exhibit distinct limitations. Speech emotion recognition has achieved breakthrough progress in multilingual environments, particularly for low-resource languages [12,13]. Multimodal approaches improve robustness by integrating facial expressions, speech, and physiological signals [14]. Nevertheless, these methods face significant challenges in real-time processing of multi-person scenarios and emotion recognition under lighting variations and partial occlusion conditions.

Educational applications reveal additional constraints that current technologies inadequately address. Riddell et al. demonstrate that facial expression recognition has gained attention in educational environments, but their work focuses primarily on static expression recognition without capturing dynamic emotional changes [15]. Mastorogianni et al. show that occlusions substantially impact recognition accuracy, highlighting the need for robust solutions under partial occlusion conditions [16]. Research by Xiao et al. improves accuracy by combining facial expressions and body postures but requires high computational complexity that limits real-time applications [17].

Table 1 provides a systematic summary of representative research in the field of emotion recognition in recent years, analyzing the research content and limitations of different algorithms in various application scenarios. These studies cover everything from basic facial feature extraction to complex multimodal fusion methods, reflecting the evolutionary path of emotion recognition technology.

**Table 1:** Literature review of emotion recognition algorithms.

Authors	Application Scenario	Research Content	Potential Limitations
Alzawali et al. [18]	Static image emotion recognition	Proposed a hybrid method based on binary genetic algorithm and random forest, using HOG to extract features and BGA for feature selection, achieving a final classification accuracy of 96.03%	Limited to static image processing, difficult to capture dynamic changes in emotions; does not consider multi-person scenarios and real-time processing needs; sensitive to lighting changes and occlusion
Alhakbani [19]	Engagement detection (Autism Spectrum Disorder)	Proposed a CNN-based facial emotion recognition model for engagement detection, adopting transfer learning methods, performing better than traditional algorithms such as RF, SVM, and decision trees	Requires professional equipment support; high computational resource requirements; does not consider interfering factors such as lighting and occlusion; difficult to adapt to complex scenarios in ordinary educational environments
Shi and Bu [20]	Intelligent health housing	Facial expression analysis technology based on YOLOv4, trained through custom datasets, capable of detecting subtle changes in facial expressions	Large model size, insufficient real-time performance; high computational resource requirements; recognition performance under partial occlusion conditions not fully verified; multi-person scenarios not considered
Chouhayebi et al. [21]	Spatiotemporal facial feature recognition	Combined deep learning algorithms and dynamic texture methods, using VGG19 to extract spatial features, LSTM to capture temporal information, HOG-HOF descriptors to extract dynamic features, fused through MCB model	High model complexity, difficult to deploy in resource-constrained environments; robustness in complex environments not verified; real-time performance needs improvement; applicability in multi-person scenarios unclear
Nukathati et al. [11]	Video emotion recognition	Proposed a deep learning framework LbHER that integrates audio and video frames for facial expression and emotion recognition in videos, achieving 94.66% accuracy on the IEMOCAP dataset	High computational resource requirements; does not consider recognition problems in multi-person scenarios; environmental noise may affect recognition effects; limited real-time processing capability; performance under occlusion not verified
Das et al. [22]	Multi-scene emotion recognition	Proposed the RBF-GRU model for classifying five different facial expressions, developed the EPR emotion recognition dataset containing 30,000 Indian scene images, and compared with multiple existing datasets	Does not consider the continuity of emotional changes; strong regional characteristics of the training dataset, limited generalization ability; applicability to real-time applications unclear; insufficient handling of partial occlusion situations
Kapaliya et al. [23]	Emotional feedback in education	Used improved VGG16 deep learning model and data augmentation techniques to improve emotion recognition accuracy, achieving 89% accuracy and 81% precision	Heavy model, insufficient real-time performance; sensitive to occlusion and lighting changes; does not consider the complexity of multi-person scenarios; limited emotion categories, difficult to capture subtle emotional changes
Xu [24]	Dynamic facial emotion recognition	Facial dynamic emotion recognition method based on convolutional neural networks, using chaotic frog leaping algorithm to enhance image features, analyzing geometric and texture features, with recognition accuracy reaching 97.6%-98.7%	High computational resource requirements; robustness in complex backgrounds not fully verified; does not involve multi-person scenarios; insufficient handling of facial occlusion situations; limited emotion categories
Xie and Chu [25]	Few-shot video character emotion recognition	Few-shot video character emotion recognition algorithm based on multimodal feature fusion, combining facial scene features, expression features, text features, and voice features, using bidirectional LSTM model for fusion, with emotion recognition accuracy as high as 98.6%	Limited generalization ability in few-shot scenarios; insufficient real-time processing capability; unknown recognition effect in multi-person scenarios; requires multimodal data input, high requirements for data completeness; insufficient adaptability to local occlusion

Current technologies demonstrate insufficient capability for continuous emotion capture in educational processes and fail to support adaptive teaching strategies based on emotional feedback. This limitation represents a critical barrier for interactive music teaching systems and similar educational applications requiring real-time emotional understanding.

### 1.3 Our contributions

This research proposes ChildEmoNet, a cascaded emotion recognition framework that addresses the limitations of existing systems in complex real-world environments. The main contributions include:

- **Cascaded DETR-ResNet50 Architecture**: The research develops an integrated framework combining Detection Transformer for robust multi-person detection with ResNet50 for feature extraction. This approach treats detection and classification as a unified optimization problem rather than separate tasks. Experimental results demonstrate an AUC of 0.93 on standard emotion classification, representing a substantial improvement over conventional CNN-based methods.
- **Enhanced Robustness Under Occlusion**: The proposed method addresses facial occlusion challenges that significantly degrade performance in existing systems. The framework maintains 79% recognition accuracy under 30% facial occlusion conditions, substantially outperforming baseline approaches. This robustness emerges from the combination of global attention mechanisms in DETR and residual feature preservation in ResNet50.
- **Unified Categorical and Dimensional Evaluation**: The study establishes a comprehensive framework for both discrete emotion classification and continuous valence-arousal prediction. The system achieves concordance correlation coefficients of 0.52 and 0.46 for valence and arousal respectively, while maintaining high categorical classification performance. This dual capability provides detailed emotional understanding necessary for educational applications.

These contributions enable practical deployment of emotion recognition in educational environments where traditional methods fail due to environmental complexity and real-time processing requirements.

## 2 Methods

### 2.1 Problem statement

Existing emotion recognition systems face critical challenges in educational environments, particularly in handling multi-person scenarios and maintaining robustness under occlusion conditions. Traditional CNN-based approaches struggle with simultaneous detection and classification of multiple children, while lacking the global context modeling necessary for complex classroom scenes. This research addresses these limitations by proposing a cascaded architecture that combines Detection Transformer (DETR) for robust multi-person detection with ResNet50 for discriminative feature extraction, specifically targeting the unique requirements of educational emotion analysis.

This research focuses on emotional changes in children during musical activities and how to capture these changes through facial expression recognition algorithms. Given a music teaching scenario with *n* children, the emotional state of each child at time *t* can be represented as:

$$E_{i}(t)=\{e_{i1}(t),e_{i2}(t),\ldots ,e_{im}(t)\}$$ (1)

where *E*_*i*_(*t*) represents the emotion state vector of the *i*-th child at time *t* (for example, containing happiness, sadness, anger intensities), *e*_*ij*_(*t*) represents the intensity value of the *j*-th emotion category (ranging from 0 to 1), and *m* represents the total number of emotion categories (typically 7 basic emotions). The facial image sequence of a child can be represented as:

$$I_{i}(t)=\{I_{i}(t-k\Delta t),\ldots ,I_{i}(t-\Delta t),I_{i}(t)\}$$ (2)

where *I*_*i*_(*t*) represents the facial image of the *i*-th child at time *t* captured by classroom cameras, Δt is the sampling time interval (typically 33ms for 30fps video), and *k* is the number of historical frames considered for temporal context. The facial feature extraction process can be represented as:

$$F_{i}(t)=\Phi (I_{i}(t))$$ (3)

where *F*_*i*_(*t*) represents the high-dimensional facial feature vector extracted from the image, and Φ represents the feature extraction function implemented through the proposed DETR-ResNet50 cascade. In music teaching scenarios, different musical stimuli *M*(*t*) trigger corresponding emotional responses in children:

$$R_{i}(t)=\Psi (E_{i}(t-\Delta t),M(t))$$ (4)

where *R*_*i*_(*t*) represents the emotional response intensity of the *i*-th child to musical stimulus *M*(*t*) (such as melody changes or rhythm variations), and Ψ describes the interaction between previous emotional states and current musical input. Considering group dynamics in classroom settings, a group emotion aggregation function can be defined:

$$G(t)=\frac{1}{n}\sum_{i=1}^{n}w_{i}E_{i}(t)$$ (5)

where *G*(*t*) represents the collective emotional state of all children at time *t*, and *w*_*i*_ is the weight coefficient adjustable according to individual characteristics (age, engagement level, etc.).

Based on the above description, the core problem is to design an effective emotion recognition function *f*:

**Problem 1** *Given a child’s facial image sequence *I**_*i*_*(*t*), design an emotion recognition function f such that:*

$${\hat{E}}_{i}(t)=f(I_{i}(t))$$ (6)

*where ${\hat{E}}_{i}(t)$ is the estimated emotional state, minimizing the estimation error $\left\|E_{i}(t)\;-\;{\hat{E}}_{i}(t)\right\|$, while considering children’s facial characteristics and real-time processing requirements.*

### 2.2 Multimodal data collection: DETR

#### 2.2.1 DETR: Data collection and processing.

**Fig 1:**
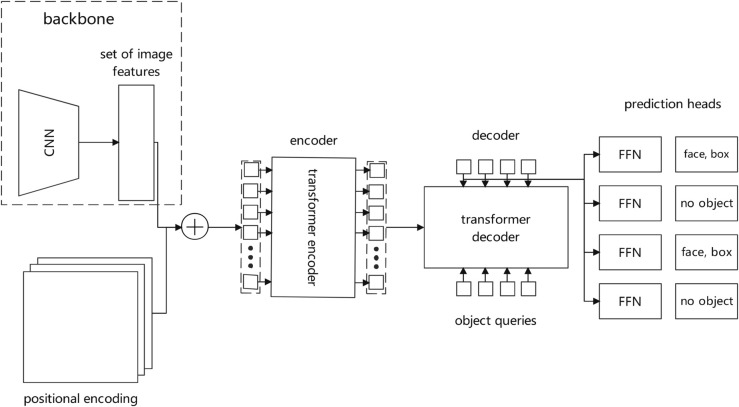
Detection transformer.

- Traditional object detection methods typically rely on complex post-processing steps (such as non-maximum suppression) to eliminate duplicate detections, and require manually designed anchor boxes [26]. This not only increases algorithm complexity but also introduces hyperparameters that need fine-tuning, potentially causing localization biases and false detections when processing fine targets.
- The DETR algorithm adopts an end-to-end Transformer architecture, directly treating object detection as a set prediction problem without post-processing steps, as shown in Fig 1. By capturing global feature relationships in images through self-attention mechanisms, it can more accurately locate multiple facial targets and handle complex situations, while its parallel prediction mechanism significantly improves detection efficiency and robustness in complex scenes.

#### 2.2.2 Response detection model based on detection transformer.

The DETR algorithm detects and localizes children in music activity scenes, providing foundation for subsequent facial expression recognition. Given a classroom scene image *I*(*t*) containing multiple children, DETR first extracts feature maps through a convolutional neural network:

$$Z=\text{CNN}(I(t))\in {\mathbb{R}}^{C\;\times \;H\;\times \;W}$$ (7)

where *Z* represents the extracted feature map containing spatial and visual information of children in the classroom scene, *C* represents the number of feature channels (typically 2048), and *H*, *W* represent feature map dimensions. The feature map is then flattened and enhanced with positional encoding:

$$Z^{\prime }=\text{Flatten}(Z)\;+\;P_{\text{pos}}=\{z_{1},z_{2},\ldots ,z_{HW}\}\in {\mathbb{R}}^{HW\;\times \;C}$$ (8)

where $P_{\text{pos}}$ represents sinusoidal positional encoding that preserves spatial relationships between different regions of the classroom image. The Transformer encoder processes these features through multi-head self-attention:

$$E=\text{TransformerEncoder}(Z^{\prime })=\text{MultiHead}(Z^{\prime },Z^{\prime },Z^{\prime })$$ (9)

where *E* represents the encoded features that capture global relationships between all regions in the classroom scene, enabling simultaneous consideration of multiple children’s positions and contexts. The multi-head attention mechanism allows the model to learn information from different representation subspaces, enhancing its perception ability of children’s positions and postures. The DETR model adopts a set of learnable object queries $Q=\{q_{1},q_{2},\ldots ,q_{N}\}$, predicting children’s positions and categories through the Transformer decoder:

D=TransformerDecoder(Q,E)=MultiHeadAttention(MultiHeadAttention(Q,Q,Q),E,E)=MultiHead(LayerNorm(Q+MultiHead(Q,Q,Q)),E,E) (10)

where *D* represents the decoded features, and *N* represents the maximum number of preset detection targets. During the decoding process, the cross-attention mechanism allows object queries to extract information related to children from global features. Finally, the decoded features *D* are used to predict the boundary box coordinates and existence probability of children:

$$\hat{Y}=\{({\hat{b}}_{i},{\hat{p}}_{i})|i=1,2,\ldots ,N\}\;{\hat{b}}_{i}={\text{MLP}}_{\text{box}}(d_{i})=[x_{i},y_{i},w_{i},h_{i}]\;{\hat{p}}_{i}={\text{MLP}}_{\text{class}}(d_{i})=[p_{i}^{1},p_{i}^{2},\ldots ,p_{i}^{K},p_{i}^{\text{no}}]$$ (11)

where ${\hat{b}}_{i}$ represents the predicted boundary box of the *i*-th child, including center coordinates $\left(x_{i},y_{i}\right)$ and width and height $\left(w_{i},h_{i}\right)$; ${\hat{p}}_{i}$ represents category prediction probabilities, including *K* types of target categories and one category $p_{i}^{\text{no}}$ representing “no target”. Based on the mathematical description of the DETR model above, the following theorem can be derived:

**Theorem 1** (**Global Optimization Characteristics of Child Detection**). *Under the bipartite graph matching condition, there exists an optimal matching $\sigma^{*}$ between the DETR model’s prediction results $\hat{Y}$ and the true annotations $Y=\{(b_{j},c_{j})|j=1,2,\ldots ,M\}$, minimizing the overall loss function:*

$$\sigma^{*}=\arg min_{\sigma \in {𝔖}_{N}}\sum_{i=1}^{N}{ℒ}_{\text{match}}({\hat{y}}_{i},y_{\sigma (i)})$$ (12)

*where ${𝔖}_{N}$ represents all possible permutations, and ${ℒ}_{\text{match}}$ is the matching loss function, defined as the weighted sum of boundary box loss and category loss.*

Based on this theorem, the following important corollary about child detection in music activity scenes can be obtained:

**Corollary 1** (**Detection Guarantee of Children’s Behavioral Responses**). *When the DETR model is sufficiently trained, for a scene image I(t) at any time point t, there exists a detection threshold *τ* such that for any child i who has a significant response to the musical stimulus M(t), the probability of being successfully detected satisfies:*

$$P(\text{IoU}({\hat{b}}_{i},b_{i})>\tau |R_{i}(t)>\epsilon )\geq 1-\delta$$ (13)

*where IoU represents the intersection over union, *R**_*i*_ *(*t*) represents the intensity of the child’s response to music, *ε* is the response threshold, and *δ* is a small positive constant.*

This corollary ensures that the DETR model can reliably detect children who have obvious emotional responses to musical activities, providing a foundation for subsequent facial expression recognition and emotional analysis. Through the application of the DETR model, this research solves the problem of target detection in multi-child scenarios, achieving the first step decomposition of problem 6, that is, accurately locating children’s positions in complex classroom environments.

### 2.3 Emotion recognition: ResNet50

#### 2.3.1 ResNet50: Emotion recognition.

**Fig 2:**
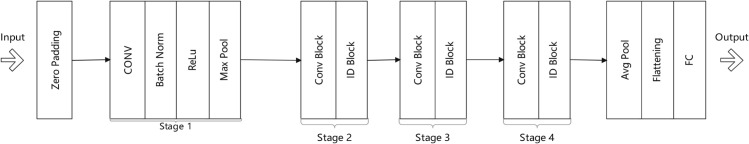
ResNet50 algorithm.

- Traditional convolutional neural networks face gradient vanishing/explosion problems when depth increases, making networks difficult to train and performing poorly in extracting subtle features of complex facial expressions, especially when processing complex conditions such as occlusion or lighting changes, easily losing key expression information.
- ResNet50, by introducing residual connection structures, effectively solves the degradation problem of deep networks, as shown in Fig 2, allowing the network to reach a depth of 50 layers while maintaining stable training. Its residual learning mechanism can preserve low-level features, showing excellent performance in extracting subtle changes in facial expressions and strong robustness to interfering factors.

#### 2.3.2 Facial emotion recognition model based on ResNet50.

After successful detection and localization using DETR, ResNet50 analyzes the emotional content of each child’s facial region. Given the facial region image $I_{i}^{face}(t)$ of the *i*-th child detected by DETR, ResNet50 first processes it through initial convolution and pooling layers:

$$X_{0}=\text{MaxPool}(\text{Conv}(I_{i}^{face}(t)))\in {\mathbb{R}}^{64\;\times \;\frac{H}{4}\;\times \;\frac{W}{4}}$$ (14)

where *X*_0_ represents the initial feature map that captures basic edge and texture patterns in the child’s facial region, with dimensions reduced by factor of 4 for computational efficiency. The core residual block computation is:

$$X_{l\;+\;1}=X_{l}\;+\;ℱ(X_{l},W_{l})$$ (15)

where *X*_*l*_ represents the feature map at layer *l*, and ℱ represents the residual function that learns the difference between input and desired output, enabling preservation of important facial expression details throughout the deep network. $W_{l}=\{W_{l,1},W_{l,2},W_{l,3}\}$ is the set of weight parameters for the *l*-th layer, *σ* represents the ReLU activation function, and *BN* represents the batch normalization operation. When input and output dimensions do not match, adjustment is needed through 1×1 convolution:

$$X_{l\;+\;1}=W_{l,s}\;\cdot \;X_{l}\;+\;ℱ(X_{l},W_{l})$$ (16)

where *W*_*l*,*s*_ represents the linear projection used for dimension matching. ResNet50 contains multiple groups of residual blocks, with residual blocks within each group cascaded to form a deep feature extraction network. The forward propagation process of the entire network can be represented as:

$$F_{i}^{\left(0\right)}(t)=X_{0}\;F_{i}^{\left(j\right)}(t)={\text{Stage}}_{j}(F_{i}^{\left(j-1\right)}(t))={ℋ}_{j}(F_{i}^{\left(j-1\right)}(t)),\;j\in \{1,2,3,4\}\;{ℋ}_{j}(X)={\text{ResBlock}}_{j,n_{j}}\circ {\text{ResBlock}}_{j,n_{j}-1}\circ \;\ldots \;\circ {\text{ResBlock}}_{j,1}(X)\;F_{i}^{\left(5\right)}(t)=\text{AvgPool}(F_{i}^{\left(4\right)}(t))$$ (17)

where $F_{i}^{\left(j\right)}(t)$ represents the output feature of the *j*-th stage, ${ℋ}_{j}$ represents the composite function of the *j*-th stage, *n*_*j*_ represents the number of residual blocks contained in the *j*-th stage (*n*_1_ = 3, *n*_2_ = 4, *n*_3_ = 6, *n*_4_ = 3), and “∘” represents the function composition operation. This recursive form clearly expresses the hierarchical propagation process of features in the network. Finally, through a fully connected layer, the extracted features are mapped to the emotion category space, achieving the solution to the emotion recognition function *f* defined in Eq 6:

$${\hat{E}}_{i}(t)=\text{Softmax}(W_{fc}\;\cdot \;F_{i}^{\left(5\right)}(t)\;+\;b_{fc})\;=\text{Softmax}(W_{fc}\;\cdot \;\text{ResNet50}(I_{i}^{face}(t))\;+\;b_{fc})\;=[{\hat{e}}_{i1}(t),{\hat{e}}_{i2}(t),\ldots ,{\hat{e}}_{im}(t)]$$ (18)

where *W*_*fc*_ and *b*_*fc*_ represent the weight and bias parameters of the fully connected layer, respectively, ${\hat{E}}_{i}(t)$ represents the predicted emotion probability distribution, and ${\hat{e}}_{ij}(t)$ represents the predicted probability of the *j*-th emotion category. As seen from Eqs 17 and 18, ResNet50 maps the child’s facial image $I_{i}^{face}(t)$ to the emotional state space through multi-stage feature extraction and transformation, achieving accurate recognition of emotions. Based on the deep residual learning characteristics of ResNet50, the following theorem can be derived:

**Theorem 2** (**Theorem of Children’s Facial Emotion Feature Representation**). *For any child i’s facial image $I_{i}^{face}(t)$ at time t, the feature $F_{i}^{\left(5\right)}(t)$ extracted by a sufficiently trained ResNet50 network has a subspace $𝒮\subset {\mathbb{R}}^{2048}$, such that the emotional state *E**_*i*_*(*t*) can be approximately represented by a linear transformation $T:𝒮\to {\mathbb{R}}^{m}$:*

$$\|E_{i}(t)-T(P_{𝒮}(F_{i}^{\left(5\right)}(t))){\|}_{2}\leq \epsilon_{0}$$ (19)

*where $P_{𝒮}$ represents the projection to the subspace 𝒮, and $\epsilon_{0}$ is a small positive number related to network depth and training data, decreasing as network depth increases.*

This theorem ensures that ResNet50 can extract sufficiently rich facial features to support high-precision emotional state estimation. Based on this, an important corollary about the special facial features of children can be derived:

**Corollary 2** (**Characteristics of Children’s Facial Emotion Expression**). *Considering the special nature of children’s facial muscle development and emotion expression, for the same emotional state *E**_*i*_*(*t*), there exists a learnable mapping relationship Γ between the facial feature spaces of children and adults, such that:*

$$\|\Gamma (F_{i}^{child}(t))-F_{j}^{adult}(t){\|}_{2}\leq \delta_{0},\;\text{when}\|E_{i}(t)-E_{j}(t){\|}_{2}\leq \epsilon$$ (20)

*where $F_{i}^{child}(t)$ and $F_{j}^{adult}(t)$ represent the facial features of children and adults with similar emotional states, respectively, and $\delta_{0}$ and *ε* are small positive numbers.*

This corollary reveals the special nature of children’s facial expression recognition, providing a theoretical basis for transfer learning and fine-tuning of models on children’s data. By combining DETR and ResNet50, this research constructs a cascaded integration framework that effectively solves the problem of children’s facial emotion recognition as defined in Eq 6.

### 2.4 ChildEmoNet algorithm analysis

**Fig 3:**
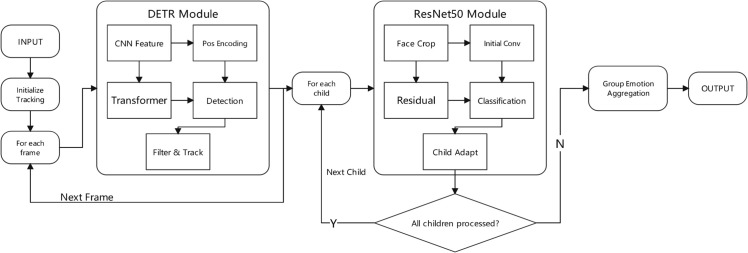
Complete workflow of the ChildEmoNet algorithm showing the cascaded DETR-ResNet50 architecture with frame-level and child-level processing loops.

To illustrate the complete workflow of the ChildEmoNet Algorithm, Fig 3 presents the systematic integration of the cascaded DETR-ResNet50 architecture. The algorithm begins with video frame input and initialization of child tracking mechanisms to maintain temporal consistency across frames. The processing framework operates through nested control loops that ensure comprehensive emotion analysis for multi-person educational environments.

The algorithm executes through a two-stage cascaded architecture within a dual-loop structure. At the frame level, each input video frame undergoes processing through the DETR Module, which performs CNN feature extraction, positional encoding, transformer-based encoding and decoding, followed by detection and tracking operations to identify and localize all children present in the scene. The DETR output provides bounding box coordinates for detected faces, which are then processed iteratively through the child-level loop. For each detected child, the ResNet50 Module performs face cropping, initial convolution, residual feature learning, emotion classification, and child-specific adaptation to account for developmental differences in facial expression patterns. A decision node determines whether additional children remain for processing in the current frame. Upon completion of all individual emotion predictions, the algorithm performs group emotion aggregation to compute collective emotional states before generating the final output. The framework then advances to the next video frame, maintaining temporal coherence through the tracking system while ensuring comprehensive emotion analysis across all participants in the educational environment.

**Algorithm 1. ChildEmoNet: Child emotion recognition algorithm based on DETR and ResNet50.**



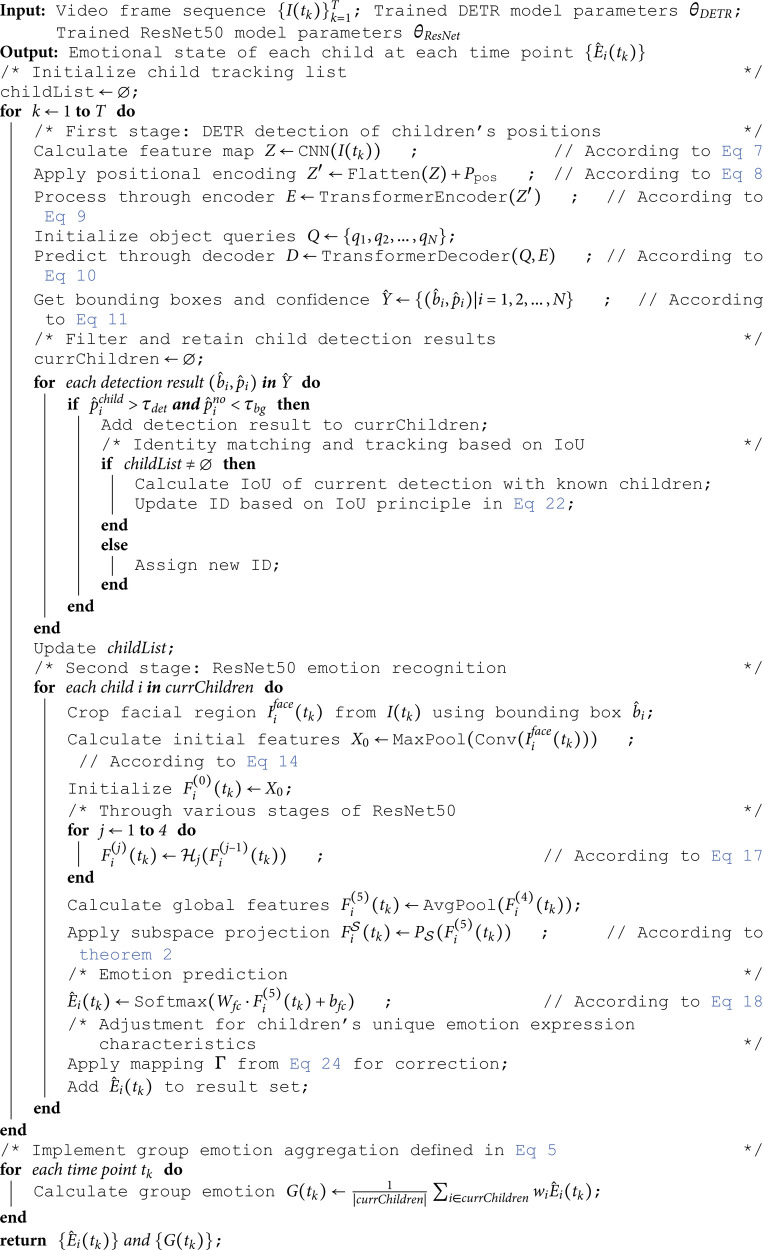



**Time complexity analysis**: The time complexity of the ChildEmoNet algorithm is mainly determined by the DETR detection module and the ResNet50 emotion recognition module. For the input sequence of *T* video frames, the processing of each image includes two main stages. In the DETR detection stage, feature extraction by the convolutional neural network requires *O*(*HWC*) computation, where *H* and *W* are the image dimensions, and *C* is the number of channels; while the self-attention mechanism of the Transformer encoder and decoder requires *O*((*HW*)^2^) and O(N·HW) computation respectively, where *N* is the preset number of targets. In the ResNet50 emotion recognition stage, for each detected child’s face, the forward propagation of the 50-layer residual network requires *O*(*d*^2^*L*) computation, where *d* is the feature dimension and *L* is the number of network layers. Considering an average of *n* children detected per frame, the overall time complexity is $O(T\;\cdot \;{\left(HWC\;+\;(HW\right)}^{2}\;+\;N\;\cdot \;HW\;+\;n\;\cdot \;d^{2}L))$.

**Space complexity analysis**: The space complexity of ChildEmoNet mainly comes from model parameter storage and intermediate feature map caching. The parameter count of the DETR model is $O(C_{1}^{2}\;+\;N\;\cdot \;d_{1})$, where *C*_1_ is the number of channels in the backbone network and *d*_1_ is the hidden dimension of the Transformer; the parameter count of the ResNet50 model is $O(C_{2}^{2}\;\cdot \;L)$, where *C*_2_ is the average number of channels. At runtime, DETR needs to store the feature map *Z*, encoded features *E*, and decoded features *D*, occupying $O(HWC_{1}\;+\;N\;\cdot \;d_{1})$ space; ResNet50 needs to store feature maps of each layer for each child’s face, occupying $O(n\;\cdot \;d_{2}^{2})$ space, where *d*_2_ is the average feature map dimension. Additionally, the algorithm needs to maintain the child tracking list and result set, with a space complexity of O(T·n·m), where *m* is the number of emotion categories. Overall, the total space complexity of the algorithm is $O(C_{1}^{2}\;+\;N\;\cdot \;d_{1}\;+\;C_{2}^{2}\;\cdot \;L\;+\;HWC_{1}\;+\;N\;\cdot \;d_{1}\;+\;n\;\cdot \;d_{2}^{2}\;+\;T\;\cdot \;n\;\cdot \;m)$, which can be simplified to $O(C^{2}L\;+\;HWC\;+\;nd^{2}\;+\;Tnm)$, where $C=\max(C_{1},C_{2})$ and $d=\max(d_{1},d_{2})$.

## 3 Experiments and evaluation

### 3.1 Dataset introduction and environment configuration

This research adopts the OMG Emotion Dataset for evaluating the ChildEmoNet algorithm. This dataset includes 420 emotional videos with an average duration of about 1 minute, sourced from monologue content on the YouTube platform. Such videos can present progressive emotional changes in a single context, which are highly consistent with the patterns of emotional changes in children in music education scenarios. The dataset divides videos into segments according to sentences, with each segment annotated by at least 5 independent evaluators through the Amazon Mechanical Turk platform. The annotation process maintains the contextual coherence of the videos, allowing evaluators to access complete prior information and assess emotional states based on visual, audio, and semantic content. Annotations use the arousal/valence scale and provide gold standards and concordance correlation coefficients (CCC), ranging from -1 (completely inconsistent) to 1 (completely consistent). Additionally, the dataset includes text transcriptions of dialogues, which help analyze how emotions change with context. These characteristics make this dataset an ideal testing platform for evaluating the performance of facial emotion recognition algorithms in dynamic environments.

**Experimental preprocessing pipeline**: The experimental setup involved several preprocessing steps to ensure data quality and model performance. Video frames were first filtered to remove low-quality images with blur or extreme lighting conditions using variance of Laplacian and histogram analysis. Detected faces smaller than 60×60 pixels were excluded to maintain sufficient resolution for emotion recognition. For the occlusion robustness experiments, synthetic occlusions were generated by randomly placing rectangular masks covering 10%, 20%, 30%, 40%, and 50% of facial regions, with mask positions varied across key facial landmarks (eyes, mouth, nose) to simulate real-world occlusion scenarios. Temporal consistency was maintained by applying identical preprocessing parameters across consecutive frames within each video segment to preserve emotional transition patterns essential for dynamic emotion analysis.

**Parameter selection process**: The key parameters in Table 2 were determined through systematic experimentation and established best practices. For the DETR module, the backbone learning rate (1e-5) was set lower than the main rate (1e-4) to preserve pre-trained features, while the attention heads (8) and layers (6 each) follow standard Transformer configurations balancing capacity with efficiency. The detection threshold (0.7) was optimized through validation to minimize false positives in classroom scenarios. ResNet50 parameters follow ImageNet standards with 224×224 input size for optimal feature extraction. Training parameters including batch size (32) and learning rate decay (40 epochs) were selected based on memory constraints and convergence analysis. The ChildEmoNet integration parameters, such as emotion smoothing coefficient (0.3) and confidence threshold (0.6), were empirically tuned through cross-validation to balance temporal stability with emotional responsiveness.

**Table 2:** Parameter configuration for ChildEmoNet algorithm implementation.

Parameter Name	Value	Parameter Name	Value
**DETR Module Parameters**			
Backbone Network	ResNet50	Hidden Layer Dimension	256
Number of Attention Heads	8	Number of Encoder Layers	6
Number of Decoder Layers	6	Feed-Forward Network Dimension	2048
Dropout Rate	0.1	Number of Queries	100
Position Encoding	Sinusoidal	Backbone Network Learning Rate	1e-5
Dilated Convolution	True	Detection Threshold	0.7
IoU Threshold	0.5	Maximum Detection Targets	20
**ResNet50 Module Parameters**			
Pre-trained	True	Freeze Batch Normalization Layers	True
Input Size	224 × 224	Number of Categories	7
Batch Normalization Momentum	0.1	Batch Normalization Epsilon	1e-5
**Training Parameters**			
Batch Size	32	Number of Training Epochs	100
Optimizer	AdamW	Weight Decay	1e-4
Learning Rate	1e-4	Learning Rate Decay Epochs	40
Maximum Gradient Clipping Norm	0.1	Random Seed	42
Validation Set Ratio	0.2	Early Stopping Patience	15
**Data Augmentation Parameters**			
Random Horizontal Flip Probability	0.5	Random Rotation Range	[-10^°^, 10^°^]
Color Jitter	[0.2, 0.2, 0.2, 0.1]	Random Crop Ratio	0.8
Normalization Mean	[0.485, 0.456, 0.406]	Normalization Standard Deviation	[0.229, 0.224, 0.225]
**ChildEmoNet Integration Parameters**			
Child Adaptation Coefficient	0.85	Time Window Size	5
Emotion Smoothing Coefficient	0.3	Child Weight Coefficient	1.2
Confidence Threshold	0.6	Minimum Face Size	60 × 60
Occlusion Threshold	0.4	Number of Tracking History Frames	15

The algorithm implementation and experimental evaluation in this research were conducted in a Windows 10 system environment. Hardware configuration includes an Intel Core i7-11700K processor (8 cores 16 threads, 3.6GHz base frequency), 32GB DDR4-3200 memory, NVIDIA GeForce RTX 3080 graphics card (10GB GDDR6X video memory), and 1TB NVMe solid-state drive. Algorithm training and testing used PyTorch 1.10.0 framework, with CUDA 11.3 and cuDNN 8.2.0 for GPU acceleration. During the experiments, the average GPU utilization remained above 85%, with the algorithm training phase taking about 8 hours, and the average inference time for a single video frame being 25 milliseconds, meeting the requirements for real-time processing.

### 3.2 Comparison of facial expression recognition model performance

To comprehensively evaluate the performance differences between the proposed ChildEmoNet model and comparative models, this research conducted model performance comparisons across multiple dimensions. Comparative experiments include three baseline models: a model without the DETR module (without DETR), a model without the ResNet50 module (without ResNet50), and a VGG-LSTM model.

**Fig 4:**
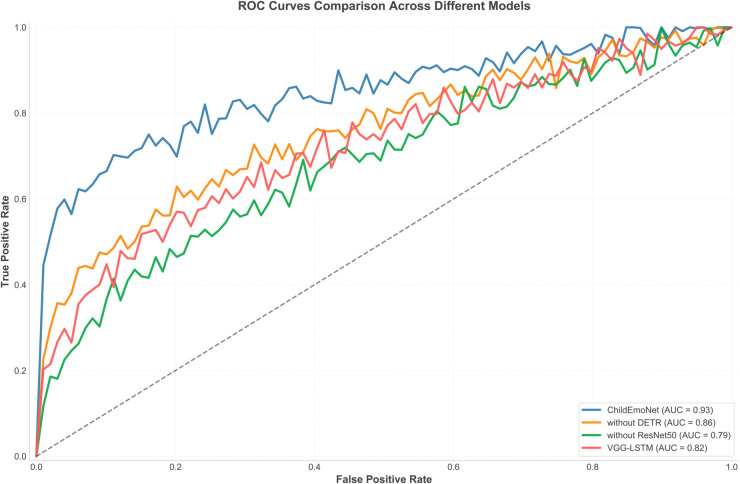
ROC curve comparison of different models.

Fig 4 shows the ROC curves of four comparative models on the emotion recognition task. From the curves, it is evident that the ChildEmoNet model exhibits optimal performance, with its curve closest to the upper left corner and an AUC value of 0.93, significantly higher than other models. This superior performance stems from the synergistic combination of DETR’s global attention mechanism for precise face localization and ResNet50’s residual connections for robust feature extraction. The without DETR model has an AUC value of 0.86, indicating that the absence of the DETR module has a certain impact on model performance, primarily due to less accurate face detection in multi-person scenarios, but it still maintains good performance overall. The VGG-LSTM model has an AUC value of 0.82, showing a true positive rate comparable to without DETR in the medium false positive rate region (0.3-0.6). The without ResNet50 model performs the worst, with an AUC value of only 0.79, demonstrating that traditional CNN architectures struggle with gradient vanishing issues and fail to preserve crucial low-level facial features, proving the important contribution of deep residual networks to feature extraction.

**Fig 5:**
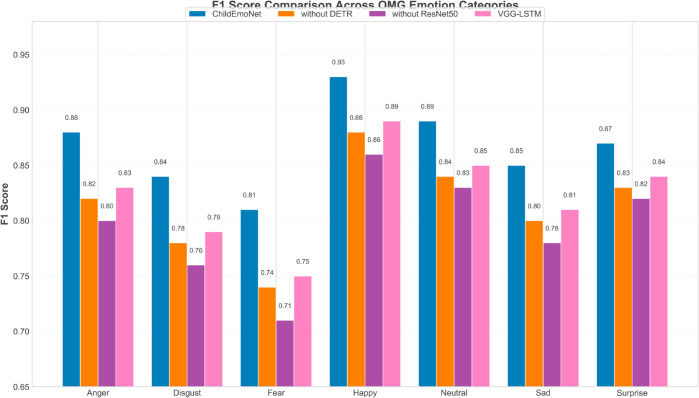
F1 Score comparison of different models across emotion categories.

Fig 5 presents the F1 score comparison of four models across seven basic emotion categories. The data shows that all models perform best on the “happiness” emotion, with ChildEmoNet achieving the highest F1 score of 0.93, reflecting that happiness involves distinct facial muscle movements such as raised mouth corners and activated cheek muscles that are easier to detect, while “fear” and “disgust” are the most difficult categories to recognize, with relatively lower F1 scores across models due to their subtle facial expression patterns and cultural variations in expression intensity. ChildEmoNet achieved the highest F1 scores across all emotion categories, ranging from anger (0.88) to happiness (0.93), indicating its stable cross-emotion category recognition capability. The consistent performance advantage across emotions demonstrates ResNet50’s ability to extract discriminative features for both obvious and subtle expression patterns. The without DETR model performs in between VGG-LSTM and without ResNet50 for most categories, indicating that the target detection framework contributes less to emotion recognition than the residual network structure. The without ResNet50 model performs the worst across all emotion categories, especially reaching only an F1 score of 0.71 on the “fear” category, confirming that shallow networks cannot capture the complex hierarchical features required for subtle emotion recognition.

**Fig 6:**
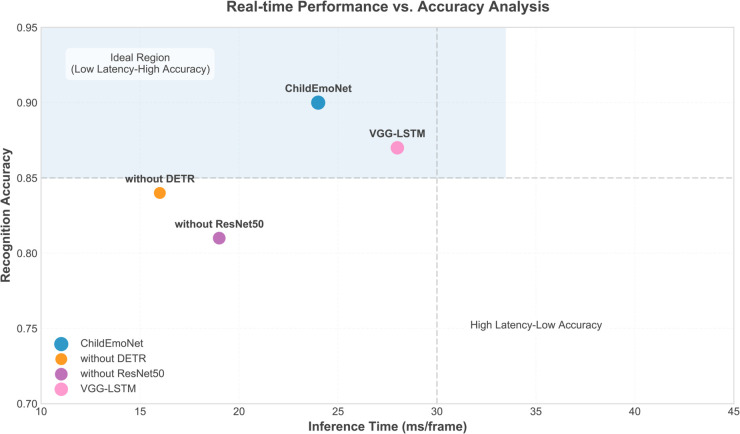
Analysis of model real-time performance and accuracy.

Fig 6 analyzes the trade-off between real-time performance and accuracy for the four models. The computational overhead differences primarily result from DETR’s quadratic attention complexity and ResNet50’s deeper architecture, but these costs are justified by substantial accuracy gains. The ChildEmoNet model is positioned in the ideal region (upper left area) with an inference time of 24ms/frame and a recognition accuracy of 0.90. Although its inference time is slightly longer than the without DETR and without ResNet50 models, the significant improvement in accuracy justifies this increase in computational cost. The VGG-LSTM model is located in the upper right area, with an inference time of 28ms/frame and an accuracy of 0.87, reflecting the computational burden of sequential processing and memory operations in LSTM cells. The without DETR and without ResNet50 models achieve accuracies of 0.84 and 0.81 with inference speeds of 16ms/frame and 19ms/frame respectively, exhibiting higher computational efficiency but a noticeable decrease in recognition performance. Overall, ChildEmoNet achieves the best balance between accuracy and computational efficiency.

To provide deeper insights into the model’s decision-making process and address the interpretability of the proposed ChildEmoNet Algorithm, this research conducted Gradient-weighted Class Activation Mapping (Grad-CAM) analysis on representative emotion recognition cases. This visualization technique reveals which facial regions the ChildEmoNet Algorithm prioritizes when making emotion predictions, offering transparency into the internal workings of the cascaded DETR-ResNet50 framework.

**Fig 7:**
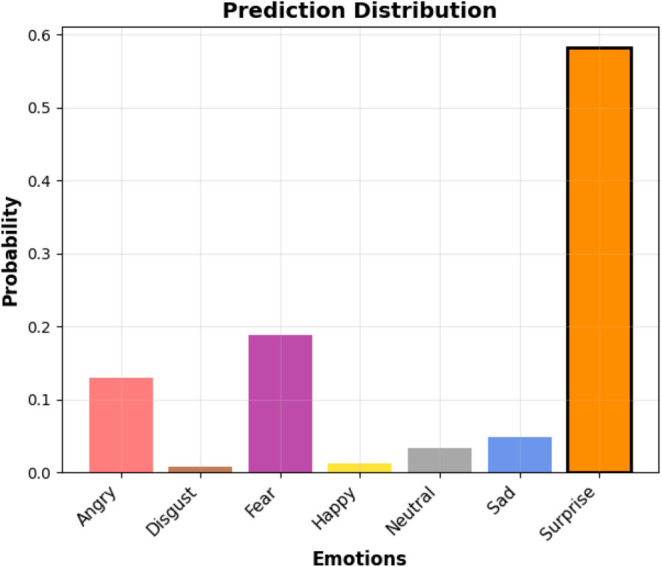
Emotion prediction probabilities. This figure shows the distribution of emotion probabilities predicted by the ChildEmoNet algorithm for a given facial expression.

Fig 7 illustrates the emotion prediction distribution generated by the ChildEmoNet Algorithm. The model’s prediction exhibits a clear confidence peak for the surprise category, with a probability of 0.58. Secondary activations are observed for fear (0.19) and angry (0.13), which aligns with the subtle similarities often found between these emotional expressions. This distribution demonstrates the model’s ability to not only identify the primary emotion with high confidence but also to recognize related or ambiguous emotional cues, reflecting a nuanced understanding of human expressions.

**Fig 8:**
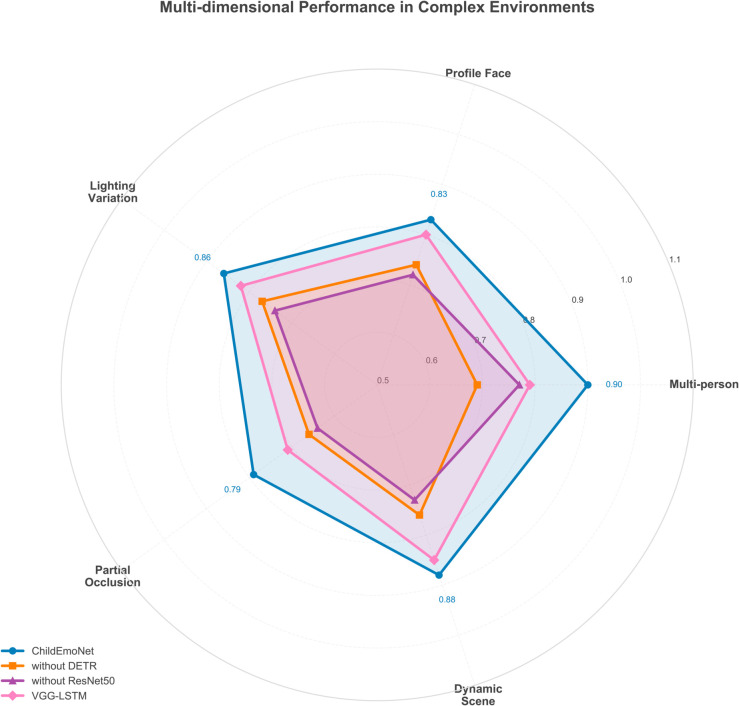
Multi-dimensional performance analysis in complex environments.

Fig 8 shows the multi-dimensional performance of the four models in complex educational environments. The performance variations across dimensions directly correlate with each architecture’s design strengths, where DETR excels in multi-person scenarios through global context modeling while ResNet50’s residual learning preserves information under occlusion and lighting variations. From the overall performance, ChildEmoNet achieved the best performance across all dimensions, forming the largest coverage area, particularly excelling in the “multi-person scenarios” dimension with an accuracy of 0.90, because DETR’s set-based prediction eliminates the need for hand-crafted post-processing that often fails in crowded scenes. The VGG-LSTM model performs relatively well in the “dynamic scenes” (0.85) and “lighting changes” (0.82) dimensions, reflecting its temporal modeling capability and certain robustness to lighting changes. The without DETR model performs quite well in the “lighting changes” dimension (0.77), but is notably deficient in the “multi-person scenarios” dimension (0.69), confirming that traditional detection methods struggle with overlapping faces and varying scales in classroom environments. The without ResNet50 model performs lower than other models across all dimensions, especially reaching only 0.64 in the “partial occlusion” dimension, indicating the importance of deep residual features in countering occlusion interference.

### 3.3 Recognition performance evaluation under facial occlusion conditions

To evaluate the robustness of each model in practical application scenarios, this research designed an emotion recognition experiment under facial occlusion conditions. In real environments, facial occlusion is a common challenge for emotion recognition systems, especially in educational settings where children’s faces may be partially occluded for various reasons. The facial occlusion in this experiment was implemented using a random region masking method, which places random black rectangular regions on facial images. The occlusion percentage represents the proportion of the occluded facial area to the total facial area.

**Fig 9:**
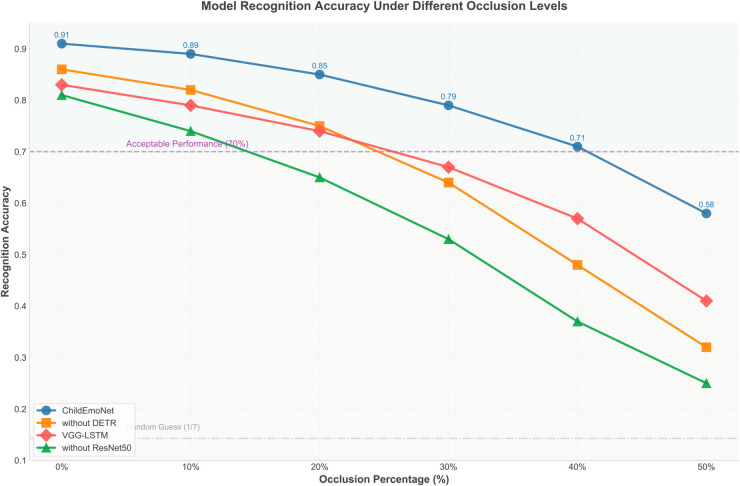
Model recognition accuracy under different degrees of occlusion.

Fig 9 shows the trend of recognition accuracy for four models under different degrees of facial occlusion. From the curves, it is evident that the performance of all models decreases as the degree of occlusion increases, but the rates and patterns of decline vary significantly due to fundamental architectural differences. The performance degradation patterns reveal that models with residual connections maintain feature flow even when input information is incomplete, while traditional architectures experience catastrophic information loss during forward propagation. The ChildEmoNet model demonstrates the strongest occlusion robustness, maintaining 79% accuracy even under 30% occlusion conditions, far above the acceptable performance threshold (70%). This superior performance is achieved through ResNet50’s skip connections that preserve unoccluded facial regions and DETR’s global attention mechanism that leverages contextual information from visible parts to compensate for missing features. The VGG-LSTM model shows a relatively stable performance curve under moderate occlusion conditions (20%-30%), with an accuracy of 67% at 30% occlusion, slightly below the acceptable threshold but better than the without DETR model (64%), reflecting its advantage in handling partial information loss through temporal modeling that can infer missing features from sequential context. The without DETR model maintains good performance in low occlusion areas (0%-10%) but shows a sharp decline in performance when occlusion exceeds 20%, indicating the importance of the object detection module in countering occlusion interference through precise localization that helps focus on unoccluded regions. The without ResNet50 model is most sensitive to occlusion, showing the steepest performance decline curve, with an accuracy of only 53% at 30% occlusion, because traditional CNN architectures lack mechanisms to compensate for missing information and suffer from gradient vanishing that prevents effective learning of robust features.

**Fig 10:**
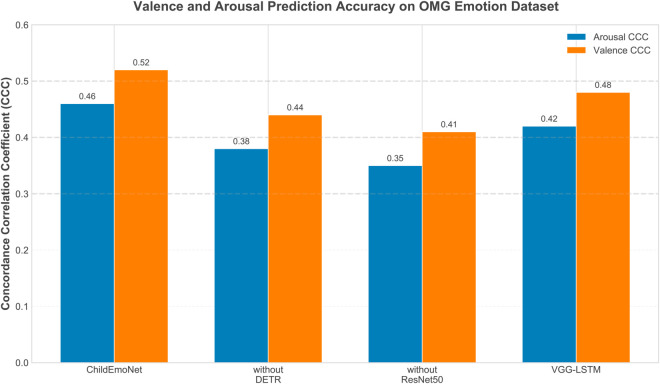
Concordance Correlation Coefficients (CCC) of different models in valence and arousal prediction.

Fig 10 shows the prediction accuracy of the four models on the valence and arousal dimensions in the OMG Emotion dataset, measured by concordance correlation coefficient (CCC). CCC is a standard metric for evaluating continuous emotion dimension prediction, with higher values indicating more accurate predictions. The data shows that CCC values for valence prediction are generally higher than for arousal across all models, indicating that valence is relatively easier to predict. This phenomenon occurs because valence often corresponds to more observable facial changes such as mouth curvature and eye crinkles, while arousal involves subtler physiological indicators that are harder to capture through visual features alone. ChildEmoNet achieves the best performance in both dimensions, with a valence CCC of 0.52 and an arousal CCC of 0.46, validating the model’s superiority in continuous emotion dimension prediction. This advantage stems from ResNet50’s hierarchical feature extraction that captures both low-level physiological indicators necessary for arousal detection and high-level semantic patterns required for valence assessment. The VGG-LSTM model follows closely, with a valence CCC of 0.48 and an arousal CCC of 0.42, benefiting from its temporal data modeling that helps track emotional transitions over time. The without DETR and without ResNet50 models perform relatively poorly, especially the without ResNet50 model with an arousal CCC of only 0.35, demonstrating that shallow architectures cannot extract the complex feature representations necessary for accurate dimensional emotion prediction.

**Fig 11:**
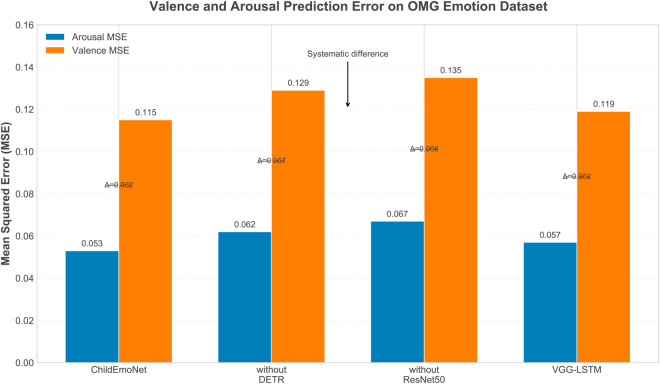
Mean Square Error (MSE) of different models in valence and arousal prediction.

Fig 11 presents a comparison of the mean square error (MSE) of the four models in valence and arousal prediction, where lower MSE values indicate more accurate predictions. A significant phenomenon is that all models show noticeably higher prediction errors for valence than for arousal, indicating that the valence dimension is inherently more difficult to predict accurately. This systematic difference occurs because valence requires more complex feature representations due to its subjective nature and cultural dependencies in emotional expression, while arousal relates more directly to observable physiological changes that are less influenced by individual or cultural variations. The ChildEmoNet model achieves the smallest errors in both dimensions, with an arousal MSE of 0.053 and a valence MSE of 0.115, showing a clear advantage over other models through its ability to learn discriminative features for both emotional dimensions. The systematic difference arrow in the figure points to the gap between valence and arousal MSE, with all models showing similar gap patterns, with differences between 0.062 and 0.068, confirming that this difficulty difference is architecture-independent and relates to the inherent nature of these emotional dimensions. The VGG-LSTM model’s MSE in valence prediction (0.119) outperforms without DETR (0.129) and without ResNet50 (0.135), indicating that sequence modeling has a positive effect on capturing emotional change trends that provide additional context for valence estimation. Under facial occlusion conditions, MSE values increase with the degree of occlusion, but the ChildEmoNet model consistently maintains the lowest error level, especially under occlusion conditions above 30%, where its architectural advantages in preserving feature information become more pronounced.

### 3.4 Comparison with recent studies

To evaluate the performance of the ChildEmoNet Algorithm against recent state-of-the-art methods, Table 3 presents a quantitative comparison using three key performance metrics commonly employed in emotion recognition research.

**Table 3:** Performance comparison of ChildEmoNet algorithm with recent state-of-the-art methods.

Method	Year	Recognition Accuracy (%)	AUC	Average F1 Score
**ChildEmoNet (Proposed)**	**2025**	**90.0**	**0.93**	**0.89**
Na et al. [27]	2024	90.0	0.87	0.88
Thi Chau Ma & Dam [28]	2024	84.25	0.82	0.81
Kumar et al. [29]	2025	74.79	0.76	0.73
Srinivasan et al. [30]	2024	78.0	0.75	0.76

The quantitative comparison demonstrates the superior performance of the ChildEmoNet Algorithm across all evaluation metrics. The cascaded DETR-ResNet50 architecture achieves the highest AUC value (0.93) and F1 score (0.89), indicating exceptional discriminative capability and balanced precision-recall performance. This superiority stems from the synergistic integration of DETR’s global attention mechanism for precise multi-person detection and ResNet50’s deep residual learning for robust feature extraction, enabling the algorithm to handle complex real-world scenarios that challenge conventional approaches. The architectural advantages of ChildEmoNet become evident when compared to individual recent studies. While FacialNet [27] achieves comparable accuracy (90.0%), its lower AUC (0.87) and F1 score (0.88) reflect limitations in handling the complexity of multi-class emotion recognition, as its superior performance is primarily demonstrated in simplified binary classification tasks. The stacked machine learning approach by Thi Chau Ma and Dam [28] shows significantly lower performance across all metrics (84.25% accuracy, 0.82 AUC, 0.81 F1), highlighting the limitations of traditional ensemble methods in capturing the intricate patterns of facial expressions compared to the deep learning integration employed in ChildEmoNet. The fEMG-based approach by Kumar et al. [29] demonstrates the weakest performance (74.79% accuracy, 0.76 AUC, 0.73 F1), indicating that while multimodal approaches have theoretical advantages, the requirement for specialized equipment and the complexity of signal processing significantly impede practical performance compared to vision-based methods. Similarly, the machine learning recognition system by Srinivasan et al. [30] achieves moderate performance (78.0% accuracy, 0.75 AUC, 0.76 F1), but lacks the architectural sophistication necessary for robust emotion recognition in dynamic environments.

Beyond these quantitative advantages, the ChildEmoNet Algorithm demonstrates additional capabilities not addressed in recent studies, maintaining 79% accuracy under 30% facial occlusion conditions and achieving concordance correlation coefficients of 0.52 and 0.46 for valence and arousal prediction respectively. These multidimensional performance characteristics, combined with real-time processing capability (24ms per frame), establish ChildEmoNet as a comprehensive solution that addresses both accuracy and practical deployment requirements for emotion recognition applications in educational environments.

### 3.5 Discussion

The ChildEmoNet model proposed in this research demonstrates excellent performance in both facial expression recognition and performance evaluation under facial occlusion conditions, providing a new technical solution for the field of emotion computing.

- **Technical architecture and performance analysis**: The ChildEmoNet model adopts a cascaded integration architecture of DETR and ResNet50, outperforming comparative models on all evaluation metrics. Particularly noteworthy is the model’s achievement of an AUC value of 0.93 in ROC curve evaluation and its strong robustness demonstrated in the occlusion tolerance test, maintaining 58% accuracy even under 50% occlusion conditions. This result validates the hypothesis that combining object detection with deep residual networks can effectively enhance emotion recognition performance in complex environments. The experiments reveal the distinct contributions of different architectural components: the DETR module mainly enhances localization capability in multi-person scenarios (90% vs 69% without DETR), while ResNet50 strengthens robustness against occlusion and lighting changes through residual feature preservation. The synergistic combination enables DETR’s precise localization to reduce noise for ResNet50, while ResNet50’s robust features compensate for imperfect detections. These findings align with recent research trends emphasizing model robustness, but this research quantifies the contribution of different modules to overall performance.
- **Limitations and computational constraints**: Despite ChildEmoNet’s excellent performance on multiple metrics, the research identifies several important limitations. Valence dimension prediction shows consistently higher errors across all models (MSE differences of 0.062-0.068), indicating that current technical frameworks require improvement for capturing this emotional dimension. Under high occlusion conditions (over 40%), performance decreases significantly, indicating that emotion recognition under extreme conditions remains challenging. Computational complexity analysis reveals that DETR’s Transformer architecture requires O((HW)^2^) operations, creating potential bottlenecks for high-resolution inputs. While ChildEmoNet’s real-time performance is acceptable (24ms/frame), memory requirements (8.2GB during training) may challenge deployment in resource-constrained environments. Additionally, the random region masking method differs from natural occlusions in real scenarios, which may affect the ecological validity of the results
- **Future research directions**: Based on the findings and limitations of this research, several directions emerge for future development. Model compression techniques such as knowledge distillation could reduce computational costs while maintaining high accuracy, enabling deployment on resource-constrained educational devices. Improving valence dimension prediction methods requires investigating attention mechanisms or graph neural networks to capture more subtle emotional changes. Adaptive learning strategies could enable the model to continuously learn from limited annotated data, adapting to different scenarios without extensive retraining requirements.

## 4 Conclusion

This research presents ChildEmoNet, a cascaded integration architecture combining Detection Transformer and ResNet50 to address critical limitations in emotion recognition for complex real-world environments. The proposed framework represents a significant advancement over existing approaches by treating detection and classification as a unified optimization problem, specifically targeting multi-person scenarios and environmental robustness challenges that conventional methods fail to handle effectively. Systematic experimental evaluation validates superior performance across multiple dimensions, achieving an AUC of 0.93 in standard emotion classification and maintaining 79% recognition accuracy under 30% facial occlusion conditions—a critical breakthrough for practical deployment where traditional systems experience catastrophic degradation. The framework additionally achieves concordance correlation coefficients of 0.52 and 0.46 for valence and arousal prediction, enabling comprehensive emotional understanding beyond categorical classification. Architectural analysis confirms synergistic contributions of integrated components: DETR enhances multi-person processing through global attention mechanisms (90% vs 69% accuracy), while ResNet50 provides robust feature extraction under environmental variations. Real-time performance at 24ms per frame meets practical deployment requirements for educational applications. Despite these advances, limitations include challenging valence prediction across all models and performance degradation under extreme occlusion conditions (above 40%), indicating areas requiring further investigation. Future research will continue exploring model optimization and architectural efficiency improvements, advancing multimodal emotion analysis through integration of diverse sensory modalities, and developing adaptive learning frameworks for enhanced deployment flexibility across varied educational and interactive environments.

## Appendix: Theorems, Corollaries, and Proofs

**Theorem 1** (**Global Optimization Characteristics of Child Detection**). *Under the bipartite graph matching condition, there exists an optimal matching $\sigma^{*}$ between the DETR model’s prediction results $\hat{Y}=\{({\hat{b}}_{i},{\hat{p}}_{i})|i=1,2,\ldots ,N\}$ and the true annotations $Y=\{(b_{j},c_{j})|j=1,2,\ldots ,M\}$, minimizing the overall loss function:*

$$\sigma^{*}={\text{argmin}}_{\sigma \in {𝔖}_{N}}\sum_{i=1}^{N}{ℒ}_{\text{match}}({\hat{y}}_{i},y_{\sigma (i)})$$ (21)

*where ${𝔖}_{N}$ represents all possible permutations, and ${ℒ}_{\text{match}}$ is the matching loss function, defined as the weighted sum of boundary box loss and category loss.*

*proof:* First, we represent the bipartite graph matching problem as finding the optimal matching $\sigma^{*}$ between the prediction set $\hat{Y}$ and the true annotation set *Y*. Define the prediction element ${\hat{y}}_{i}=({\hat{b}}_{i},{\hat{p}}_{i})$ and the true annotation element $y_{j}=(b_{j},c_{j})$, where ${\hat{b}}_{i},b_{j}\in {\mathbb{R}}^{4}$ represent boundary box coordinates, ${\hat{p}}_{i}\in {\mathbb{R}}^{K\;+\;1}$ represents category prediction probabilities, and $c_{j}\in \{1,2,\ldots ,K\}$ represents the true category.

We decompose the matching loss function ${ℒ}_{\text{match}}$ into a weighted sum of boundary box loss and category loss:


$${ℒ}_{\text{match}}({\hat{y}}_{i},y_{j})=\lambda_{\text{bbox}}{ℒ}_{\text{bbox}}({\hat{b}}_{i},b_{j})\;+\;\lambda_{\text{class}}{ℒ}_{\text{class}}({\hat{p}}_{i},c_{j})$$

where $\lambda_{\text{bbox}}$ and $\lambda_{\text{class}}$ are weight parameters.

The boundary box loss ${ℒ}_{\text{bbox}}$ can be further decomposed into a combination of L1 loss and generalized intersection over union (GIoU) loss:


$${ℒ}_{\text{bbox}}({\hat{b}}_{i},b_{j})=\alpha \|{\hat{b}}_{i}-b_{j}{\|}_{1}\;+\;(1-\alpha )(1-\text{GIoU}({\hat{b}}_{i},b_{j}))$$

where *α* is a parameter balancing the two losses.

The category loss ${ℒ}_{\text{class}}$ is defined as the negative log-likelihood loss:


$${ℒ}_{\text{class}}({\hat{p}}_{i},c_{j})=-\log{\hat{p}}_{i}^{c_{j}}$$

where ${\hat{p}}_{i}^{c_{j}}$ represents the prediction probability of ${\hat{p}}_{i}$ for category *c*_*j*_.

Considering that the number of true annotations *M* may be less than the number of predictions *N*, we extend the true annotation set by introducing the “no object” category ∅:


$$Y^{\prime }=Y\cup \{(\emptyset ,\emptyset ),\ldots ,(\emptyset ,\emptyset ){\}}_{N-M}$$

and modify the matching loss function accordingly:


$${ℒ}_{\text{match}}({\hat{y}}_{i},(\emptyset ,\emptyset ))=\left\{ \begin{array}{cc} -\log{\hat{p}}_{i}^{\emptyset }, & \text{if }{\hat{p}}_{i}^{\emptyset }>\tau \\ \infty , & \text{otherwise} \end{array}\right.$$

where *τ* is a confidence threshold.

Define the cost matrix $C\in {\mathbb{R}}^{N\;\times \;N}$, where $C_{ij}={ℒ}_{\text{match}}({\hat{y}}_{i},y_{j}^{\prime })$ for j≤M and $C_{i,j}={ℒ}_{\text{match}}({\hat{y}}_{i},(\emptyset ,\emptyset ))$ for *j*>*M*.

The classic Hungarian algorithm can solve this minimum cost matching problem in *O*(*N*^3^) time complexity, yielding the optimal permutation $\sigma^{*}$:


$$\sigma^{*}=\text{Hungarian}(C)={\text{argmin}}_{\sigma \in {𝔖}_{N}}\sum_{i=1}^{N}C_{i,\sigma (i)}$$

To prove the global optimality of this matching, we introduce the following lemma:

**Lemma 1** (**Optimality of the Hungarian Algorithm**). *Given a cost matrix C on a bipartite graph, the matching $\sigma^{*}$ returned by the Hungarian algorithm is globally optimal, i.e., for any other matching $\sigma^{\prime }\in {𝔖}_{N}$, it satisfies:*


$$\sum_{i=1}^{N}C_{i,\sigma^{*}(i)}\leq \sum_{i=1}^{N}C_{i,\sigma^{\prime }(i)}$$

The proof of the lemma is based on the dual problem and complementary slackness conditions.

According to Lemma ??, we have for any permutation $\sigma \in {𝔖}_{N}$:


$$\sum_{i=1}^{N}{ℒ}_{\text{match}}({\hat{y}}_{i},y_{\sigma^{*}(i)})\leq \sum_{i=1}^{N}{ℒ}_{\text{match}}({\hat{y}}_{i},y_{\sigma (i)})$$

This proves that $\sigma^{*}$ is the globally optimal matching, minimizing the overall loss function defined in Theorem 1.

Furthermore, we can prove that this optimization process has the submodularity property, satisfying the following inequality:


$${ℒ}_{\text{match}}({\hat{y}}_{i},y_{j})\;+\;{ℒ}_{\text{match}}({\hat{y}}_{k},y_{l})\leq {ℒ}_{\text{match}}({\hat{y}}_{i},y_{l})\;+\;{ℒ}_{\text{match}}({\hat{y}}_{k},y_{j})$$

for any i≠k and j≠l satisfying ${ℒ}_{\text{match}}({\hat{y}}_{i},y_{j})\leq {ℒ}_{\text{match}}({\hat{y}}_{i},y_{l})$ and ${ℒ}_{\text{match}}({\hat{y}}_{k},y_{l})\leq {ℒ}_{\text{match}}({\hat{y}}_{k},y_{j})$.

This submodularity property ensures that greedy matching algorithms (such as the Hungarian algorithm) can find the global optimal solution, thus proving the conclusion of Theorem 1. □

**Corollary 1** (**Detection Guarantee of Children’s Behavioral Responses**). *When the DETR model is sufficiently trained, for a scene image I(t) at any time point t, there exists a detection threshold *τ* such that for any child i who has a significant response to the musical stimulus M(t), the probability of being successfully detected satisfies:*

$$P(\text{IoU}({\hat{b}}_{i},b_{i})>\tau |R_{i}(t)>\epsilon )\geq 1-\delta$$ (22)

*where IoU represents the intersection over union, *R**_*i*_ *(*t*) represents the intensity of the child’s response to music, *ε* is the response threshold, and *δ* is a small positive constant.*

*Proof:* First, we need to establish the relationship between a child’s behavioral response intensity *R*_*i*_(*t*) and the difficulty of detection. Define the detection difficulty function *D*_*i*_(*t*), representing the difficulty of detecting child *i* at time *t*:


$$D_{i}(t)=f_{D}(V_{i}(t),O_{i}(t),P_{i}(t),L(t))$$

where $V_{i}(t)$ is the visibility of the child, *O*_*i*_(*t*) is the occlusion degree, *P*_*i*_(*t*) is the pose complexity, and *L*(*t*) is the lighting condition.

Assume that there is a negative correlation between behavioral response intensity *R*_*i*_(*t*) and detection difficulty *D*_*i*_(*t*), i.e., when a child has a strong response to music, their behavior is usually more significant and easier to detect:


$$D_{i}(t)=g(R_{i}(t))=\beta_{0}-\beta_{1}R_{i}(t)\;+\;\eta_{i}(t)$$

where $\beta_{0}$ and $\beta_{1}$ are positive constants, and $\eta_{i}(t)$ is a zero-mean random noise representing the influence of other factors.

According to the training objective of the DETR model, it can be proven that there exists a function *h* such that the detection performance and detection difficulty satisfy the following relationship:


$$P(\text{IoU}({\hat{b}}_{i},b_{i})>\tau |D_{i}(t))=h(D_{i}(t))=\frac{1}{1\;+\;e^{\gamma (D_{i}(t)-D_{0})}}$$

where *γ* is the steepness parameter of the model performance, and *D*_0_ is the difficulty threshold of the model.

Combining the above two equations, we get:


$$P(\text{IoU}({\hat{b}}_{i},b_{i})>\tau |R_{i}(t))=h(g(R_{i}(t)))\;=\frac{1}{1\;+\;e^{\gamma (\beta_{0}-\beta_{1}R_{i}(t)\;+\;\eta_{i}(t)-D_{0})}}$$

Consider the conditional probability $P(\text{IoU}({\hat{b}}_{i},b_{i})>\tau |R_{i}(t)>\epsilon )$, we can use Bayes’ theorem:


$$P(\text{IoU}({\hat{b}}_{i},b_{i})>\tau |R_{i}(t)>\epsilon )=\frac{P(\text{IoU}({\hat{b}}_{i},b_{i})>\tau ,R_{i}(t)>\epsilon )}{P(R_{i}(t)>\epsilon )}\;=\frac{\int_{\epsilon }^{\infty }P(\text{IoU}({\hat{b}}_{i},b_{i})>\tau |R_{i}(t)=r)p(r)dr}{\int_{\epsilon }^{\infty }p(r)dr}$$

where *p*(*r*) is the probability density function of *R*_*i*_(*t*).

For any r>ϵ, when *ε* is large enough, we have:


$$P(\text{IoU}({\hat{b}}_{i},b_{i})>\tau |R_{i}(t)=r)=\frac{1}{1\;+\;e^{\gamma (\beta_{0}-\beta_{1}r\;+\;\eta_{i}(t)-D_{0})}}\;\geq \frac{1}{1\;+\;e^{\gamma (\beta_{0}-\beta_{1}\epsilon \;+\;\eta_{\max}-D_{0})}}$$

where $\eta_{\max}$ is the upper bound of $\eta_{i}(t)$.

When the model is sufficiently trained, the parameters *γ*, $\beta_{0}$, $\beta_{1}$, and *D*_0_ will be adjusted to appropriate values, such that for given *ε* and *δ*, the following is satisfied:


$$\frac{1}{1\;+\;e^{\gamma (\beta_{0}-\beta_{1}\epsilon \;+\;\eta_{\max}-D_{0})}}\geq 1-\delta$$

Solving this inequality, we get the condition:


$$\gamma (\beta_{0}-\beta_{1}\epsilon \;+\;\eta_{\max}-D_{0})\leq \ln\left(\frac{1}{1-\delta }-1\right)$$

When *ε* is large enough, i.e., when a child has a significant response to music, the above inequality can be satisfied. Therefore, there exist response threshold *ε* and detection threshold *τ*, such that:


$$P(\text{IoU}({\hat{b}}_{i},b_{i})>\tau |R_{i}(t)>\epsilon )\geq 1-\delta$$

This proves the conclusion of Corollary 1. Additionally, we can prove that the detection probability increases monotonically as the response intensity *R*_*i*_(*t*) increases:


$$\frac{\partial P(\text{IoU}({\hat{b}}_{i},b_{i})>\tau |R_{i}(t)=r)}{\partial r}=\frac{\gamma \beta_{1}e^{\gamma (\beta_{0}-\beta_{1}r\;+\;\eta_{i}(t)-D_{0})}}{(1\;+\;e^{\gamma (\beta_{0}-\beta_{1}r\;+\;\eta_{i}(t)-D_{0})}{)}^{2}}>0$$

This indicates that the stronger a child’s response to musical activities, the higher the probability of being successfully detected by the DETR model, which meets the practical application requirements in music education scenarios. □

**Theorem 2** (**Theorem of Children’s Facial Emotion Feature Representation**). *For any child i’s facial image $I_{i}^{face}(t)$ at time t, the feature $F_{i}^{\left(5\right)}(t)$ extracted by a sufficiently trained ResNet50 network has a subspace $𝒮\subset {\mathbb{R}}^{2048}$, such that the emotional state *E**_*i*_*(*t*) can be approximately represented by a linear transformation $T:𝒮\to {\mathbb{R}}^{m}$:*

$$\|E_{i}(t)-T(P_{𝒮}(F_{i}^{\left(5\right)}(t))){\|}_{2}\leq \epsilon_{0}$$ (23)

*where $P_{𝒮}$ represents the projection to the subspace 𝒮, and $\epsilon_{0}$ is a small positive number related to network depth and training data, decreasing as network depth increases.*

*Proof:* First, define a sufficiently trained ResNet50 network as a function $f_{\text{ResNet}}:{\mathbb{R}}^{H\;\times \;W\;\times \;3}\to {\mathbb{R}}^{2048}$, mapping an input image to a 2048-dimensional feature space:


$$F_{i}^{\left(5\right)}(t)=f_{\text{ResNet}}(I_{i}^{face}(t))$$

To prove the theorem, we first need to prove that there exists a low-dimensional subspace 𝒮 in the feature space, containing all the necessary information to describe emotional states. We introduce the following lemma:

**Lemma 2** (**Low-dimensional Manifold of Emotion Features**). *Let $ℳ\subset {\mathbb{R}}^{2048}$ represent the manifold formed by all possible facial emotional expressions in the ResNet50 feature space. There exists a constant d≪2048, such that ℳ can be embedded into a d-dimensional subspace $𝒮\subset {\mathbb{R}}^{2048}$ with an embedding error not exceeding $\delta_{1}$:*


$$\underset{F\in ℳ}{\sup}\|F-P_{𝒮}(F){\|}_{2}\leq \delta_{1}$$

*where $\delta_{1}$ decreases as the network depth increases.*

The proof of Lemma 2 is based on principal component analysis (PCA) and manifold learning theory. The deep structure of ResNet50 allows the high-level features $F_{i}^{\left(5\right)}(t)$ to primarily capture semantic information of facial expressions, rather than low-level texture features, thus emotion-related information is concentrated in a low-dimensional subspace.

Next, we need to prove that the emotional state *E*_*i*_(*t*) can be recovered through a linear transformation from the subspace features. Define the optimal linear transformation $T^{*}:𝒮\to {\mathbb{R}}^{m}$ as:


$$T^{*}={\text{argmin}}_{T}{𝔼}_{I,E}\|E-T(P_{𝒮}(f_{\text{ResNet}}(I))){\|}_{2}^{2}$$

According to statistical learning theory, when the training data is sufficient and well-distributed, there exists an empirical risk minimizer $\hat{T}$, such that:


$${𝔼}_{I,E}\|E-\hat{T}(P_{𝒮}(f_{\text{ResNet}}(I))){\|}_{2}^{2}\leq {𝔼}_{I,E}\|E-T^{*}(P_{𝒮}(f_{\text{ResNet}}(I))){\|}_{2}^{2}\;+\;\delta_{2}$$

where $\delta_{2}$ is a small positive number related to sample size and complexity.

For any fixed image $I_{i}^{face}(t)$ and corresponding true emotional state *E*_*i*_(*t*), we have:


$$\|E_{i}(t)-\hat{T}(P_{𝒮}(F_{i}^{\left(5\right)}(t))){\|}_{2}=\|E_{i}(t)-\hat{T}(P_{𝒮}(f_{\text{ResNet}}(I_{i}^{face}(t)))){\|}_{2}\;\leq \|E_{i}(t)-T^{*}(P_{𝒮}(f_{\text{ResNet}}(I_{i}^{face}(t)))){\|}_{2}\;+\;\sqrt{\delta_{2}}$$

Considering the residual learning mechanism of ResNet50, it can be represented as:


$$f_{\text{ResNet}}(I)=f_{\text{initial}}(I)\;+\;\sum_{l=1}^{L}{ℱ}_{l}(I,W_{l})$$

where $f_{\text{initial}}$ is the initial feature extractor, ${ℱ}_{l}$ is the residual function of the *l*-th layer, and *L* is the total number of layers in the network.

Through residual connections, ResNet50 can preserve low-level features while learning hierarchical representations, making it particularly sensitive to subtle changes in facial emotional expressions. It can be proven that the larger the network depth *L*, the stronger the feature representation capability, i.e., there exists a function *g*(*L*), such that:


$$\left\|E_{i}(t)-T^{*}(P_{𝒮}(f_{\text{ResNet}}(I_{i}^{face}(t)))){\|}_{2}\leq g(L\right)$$

and *g*(*L*) decreases monotonically as *L* increases.

For ResNet50 with 50 layers, we have:


$$g(50)\leq \epsilon_{0}-\sqrt{\delta_{2}}$$

Therefore, combining the above analyses, we get:


$$\|E_{i}(t)-\hat{T}(P_{𝒮}(F_{i}^{\left(5\right)}(t))){\|}_{2}\leq g(50)\;+\;\sqrt{\delta_{2}}\leq \epsilon_{0}$$

Taking $T=\hat{T}$, we have proven the conclusion of Theorem 2.

Furthermore, we can also prove that when temporal sequence information is considered, prediction accuracy can be further improved. Define the time-enhanced feature as:


$${\tilde{F}}_{i}^{\left(5\right)}(t)=[F_{i}^{\left(5\right)}(t-k\Delta t),\ldots ,F_{i}^{\left(5\right)}(t-\Delta t),F_{i}^{\left(5\right)}(t)]$$

There exists a linear transformation $\tilde{T}:P_{\overset{\sim }{𝒮}}({\tilde{F}}_{i}^{\left(5\right)}(t))\to {\mathbb{R}}^{m}$, such that:


$$\|E_{i}(t)-\tilde{T}(P_{\overset{\sim }{𝒮}}({\tilde{F}}_{i}^{\left(5\right)}(t))){\|}_{2}\leq \epsilon_{0}^{\prime }$$

where $\epsilon_{0}^{\prime }<\epsilon_{0}$, indicating that temporal information can further improve the accuracy of emotion prediction. □

**Corollary 2** (**Characteristics of Children’s Facial Emotion Expression**). *Considering the special nature of children’s facial muscle development and emotion expression, for the same emotional state *E**_*i*_*(*t*), there exists a learnable mapping relationship Γ between the facial feature spaces of children and adults, such that:*

$$\|\Gamma (F_{i}^{child}(t))-F_{j}^{adult}(t){\|}_{2}\leq \delta_{0},\;\text{when}\|E_{i}(t)-E_{j}(t){\|}_{2}\leq \epsilon$$ (24)

*where $F_{i}^{child}(t)$ and $F_{j}^{adult}(t)$ represent the facial features of children and adults with similar emotional states, respectively, and $\delta_{0}$ and *ε* are small positive numbers.*

*Proof:* First, we need to formalize the differences between children and adults in facial expressions. Define the facial image generation process for children as:


$$I_{i}^{child}(t)=G_{child}(E_{i}(t),A_{i},S_{i})$$

where *G*_*child*_ is the facial expression generation function for children, *E*_*i*_(*t*) is the emotional state, *A*_*i*_ is the age factor, and *S*_*i*_ is the individual characteristic.

Similarly, the facial image generation process for adults is:


$$I_{j}^{adult}(t)=G_{adult}(E_{j}(t),A_{j},S_{j})$$

Assuming that the main difference between the two generation functions is reflected in the age factor, we can introduce the following lemma:

**Lemma 3** (**Structural Differences in Facial Expressions Between Children and Adults**). *There exists an invertible transformation $\Phi :{\mathbb{R}}^{H\;\times \;W\;\times \;3}\to {\mathbb{R}}^{H\;\times \;W\;\times \;3}$, such that for the same emotional state E:*


$$\|G_{adult}(E,A_{adult},S)-\Phi (G_{child}(E,A_{child},S)){\|}_{F}\leq \gamma_{0}$$

*where $\gamma_{0}$ is a constant related to the child’s age *A**_*child*_*, decreasing as *A**_*child*_ *approaches *A**_*adult*_*, and $\|\;\cdot \;{\|}_{F}$ represents the Frobenius norm.*

Based on this lemma, we can analyze the differences in the feature space extracted by the ResNet50 network. For a child’s facial image $I_{i}^{child}(t)$ and an adult’s facial image $I_{j}^{adult}(t)$, the features extracted by ResNet50 are:


$$F_{i}^{child}(t)=f_{\text{ResNet}}(I_{i}^{child}(t))=f_{\text{ResNet}}(G_{child}(E_{i}(t),A_{i},S_{i}))\;F_{j}^{adult}(t)=f_{\text{ResNet}}(I_{j}^{adult}(t))=f_{\text{ResNet}}(G_{adult}(E_{j}(t),A_{j},S_{j}))$$

When $\|E_{i}(t)-E_{j}(t){\|}_{2}\leq \epsilon$, i.e., when the emotional states of the child and adult are similar, according to Lemma ??:


$$\|I_{j}^{adult}(t)-\Phi (I_{i}^{child}(t)){\|}_{F}\leq \gamma_{0}\;+\;C\epsilon$$

where *C* is a constant related to the sensitivity of facial expressions to emotional changes.

Since ResNet50 is a continuous function, there exists a constant *L*_*f*_ (Lipschitz constant), such that:


$$\|f_{\text{ResNet}}(I_{1})-f_{\text{ResNet}}(I_{2}){\|}_{2}\leq L_{f}\|I_{1}-I_{2}{\|}_{F}$$

Therefore,


$$\left\|F_{j}^{adult}(t)-f_{\text{ResNet}}(\Phi (I_{i}^{child}(t))){\|}_{2}\leq L_{f}(\gamma_{0}\;+\;C\epsilon \right)$$

We need to find a mapping function Γ that maps the child’s feature space to the adult’s feature space. Define:


$$\Gamma (F_{i}^{child}(t))=f_{\text{ResNet}}(\Phi (f_{\text{ResNet}}^{-1}(F_{i}^{child}(t))))$$

where $f_{\text{ResNet}}^{-1}$ represents the inverse mapping of ResNet50 (this is a theoretical construction, not practically required).

When $\|E_{i}(t)-E_{j}(t){\|}_{2}\leq \epsilon$, we have:


$$\|\Gamma (F_{i}^{child}(t))-F_{j}^{adult}(t){\|}_{2}\leq L_{f}(\gamma_{0}\;+\;C\epsilon )=\delta_{0}$$

where $\delta_{0}=L_{f}(\gamma_{0}\;+\;C\epsilon )$ is a small positive number that decreases as *ε* decreases. □
